# Platelet Serotonin Levels Are Associated with Plasma Soluble Leptin Receptor Concentrations in Normoglycemic Women

**DOI:** 10.1155/2019/2714049

**Published:** 2019-05-02

**Authors:** Luis Rodrigo Cataldo, José Suazo, Pablo Olmos, Carolina Bravo, José E. Galgani, Malin Fex, J. Alfredo Martínez, José L. Santos

**Affiliations:** ^1^Department of Nutrition, Diabetes and Metabolism, School of Medicine, Pontificia Universidad Católica de Chile, Santiago, Chile; ^2^Lund University Diabetes Centre, Clinical Research Center, Lund University, Malmö, Sweden; ^3^Institute for Research in Dental Sciences, Faculty of Dentistry, Universidad de Chile, Santiago, Chile; ^4^Department of Health Sciences, Nutrition & Dietetics, Faculty of Medicine, Pontificia Universidad Católica de Chile, Santiago, Chile; ^5^Department of Nutrition, Food Sciences and Physiology, Centre for Nutrition Research, University of Navarra, Pamplona, Spain; ^6^Centro de Investigación Biomédica en Red de la Fisiopatología de la Obesidad y Nutrición (CIBERobn), Instituto de Salud Carlos III, Madrid, Spain; ^7^Navarra's Health Research Institute (IdiSNA), Pamplona, Spain; ^8^IMDEA-Food, Madrid, Spain

## Abstract

Most peripheral serotonin (5-hydroxytryptamine (5HT)) is synthetized in the gut with platelets being its main circulating reservoir. 5HT is acting as a hormone in key organs to regulate glucose and lipid metabolism. However, the relation between platelet 5HT levels and traits related to glucose homeostasis and lipid metabolism in humans remains poorly explored. The objectives of this study were (a) to assess the association between platelet 5HT levels and plasma concentration of nonesterified fatty acids (NEFAs) and some adipokines including leptin and its soluble leptin receptor (sOb-R), (b) to assess the association between platelet 5HT levels and anthropometric traits and indexes of insulin secretion/sensitivity derived from oral glucose tolerance test (OGTT), and (c) to evaluate changes in platelet 5HT levels in response to OGTT. In a cross-sectional study, 59 normoglycemic women underwent a standard 2-hour OGTT. Plasma leptin, sOb-R, total and high molecular weight adiponectin, TNF*α*, and MCP1 were determined by immunoassays. Platelet 5HT levels and NEFAs were measured before and after OGTT. The free leptin index was calculated from leptin and sOb-R measurements. Insulin sensitivity indexes derived from OGTT (HOMA-S and Matsuda ISICOMP) and plasma NEFAs (Adipose-IR, Revised QUICKI) were also calculated. Our data show that among metabolic traits, platelet 5HT levels were associated with plasma sOb-R (*r* = 0.39, *p* = 0.003, corrected *p* = 0.018). Platelet 5HT levels were reduced in response to OGTT (779 ± 237 vs.731 ± 217 ng/10^9^ platelets, *p* = 0.005). In conclusion, platelet 5HT levels are positively associated with plasma sOb-R concentrations and reduced in response to glucose intake possibly indicating a role of peripheral 5HT in leptin-mediated appetite regulation.

## 1. Introduction

Serotonin (5-hydroxytryptamine (5HT)) is mainly synthesized by enterochromaffin cells in the gut that release large amounts of this monoamine in response to mechanical/chemical stimuli related to feeding [1]. Such gut-derived 5HT is released to systemic circulation, being transported through the body mostly within platelets [1]. Given that 5HT is not able to cross the blood-brain barrier, peripheral and brain pools of this monoamine remain independent.

The peripheral 5HT pool is composed of a major circulating fraction stored in platelets and a minor fraction that is locally produced in different tissues. Adipocytes [2] and pancreatic *β*-cells [3–7] are able to synthesize, release, and sense this monoamine. 5HT signaling is transduced by a large and complex receptor family composed of fourteen subtypes [8]. Thus, locally produced or platelet-derived 5HT may theoretically act either as a hormone or as an autocrine/paracrine factor, whose diverse metabolic effects are mediated by the specific set of 5HT receptors expressed or activated in target cells [9, 10].

Peripheral 5HT appears to influence glucose and lipid metabolism in different cell types including white and brown adipocytes, *β*-cells, myocytes, and hepatocytes [6, 9–13]. 5HT infusion in humans and dogs increases plasma nonesterified fatty acids (NEFAs) and glycerol concentrations [14]. Consistently, 5HT has shown to negatively regulate lipogenesis in human preadipocytes [15]. Pharmacological studies in genetically manipulated mice are consistent with these findings, demonstrating that gut-derived 5HT increases during fasting and promotes lipolysis in adipocytes and gluconeogenesis in hepatocytes. This report suggested that peripheral 5HT acts as a multifunctional molecule for fasting adaptation in mice [16].

Additional actions of peripheral 5HT in adipocytes include the regulation of production/activity of some adipokines. 5HT in murine 3T3-L1 adipocytes reduces the expression of adiponectin and increases that of PAI1 [17]. Moreover, a recent interventional study in healthy volunteers has shown that an acute tryptophan depletion, which decreased the brain 5HT content, reduced the plasma levels of the soluble leptin receptor (sOb-R) in women [18]. The actions of leptin in the Central Nervous System (CNS) include the regulation of glucose homeostasis and appetite control. It has been described that the availability of leptin in the CNS is partially modulated by circulating sOb-R. Then, a possible regulation of peripheral 5HT on production/activity of leptin or its inhibitor sOb-R may indirectly impact on glycemic traits and eating behavior. Thus, it is important to evaluate if there is any association between peripheral 5HT levels (mostly stored in platelets) and those circulating adipokines in humans, especially leptin and sOb-R.

Parallelly, several experimental approaches have demonstrated that 5HT regulates pancreatic *β*-cell differentiation, proliferation, and function [3, 9, 19–26]. Remarkably, during gestation, the expression of genes encoding 5HT synthesis enzymes and 5HT content itself is hugely increased in *β*-cells, promoting its proliferation to face the increased insulin resistance in peripheral tissues [19, 20]. Moreover, depending on the physiological context [9, 10], 5HT positively or negatively regulates glucose-stimulated insulin secretion (GSIS) in a receptor-dependent or receptor-independent manner in various *β*-cell models and animal or human pancreatic islets [3, 4, 20, 21, 23, 24, 27]. Emphasizing a possible important role of peripheral 5HT in glucose homeostasis, a metabolomic study in humans analyzed changes in plasma metabolites after an oral glucose tolerance test (OGTT) and found that 5HT is one of the main reduced metabolites in serum after 2 h of glucose load [28], suggesting a physiological role of peripheral 5HT in insulin secretion regulation in humans.

In spite of the increasing amount of information relating circulating 5HT to glucose and lipid metabolism, the association between platelet 5HT and glucose and lipid traits has been poorly explored in humans. In the present study, we evaluated if platelet 5HT levels are associated with plasma adipokine levels, with glucose- and lipid-related metabolic traits, and with OGTT-derived insulin secretion/sensitivity indexes in healthy normoglycemic women.

## 2. Subjects and Methods

### 2.1. Subjects

A cross-sectional study was carried out in nondiabetic nulliparous, nonpregnant women (*n* = 59, age: 27 ± 6 years, BMI: 23.6 ± 3 kg/m^2^, glycemia: 77.6 ± 5.9 mg/dl (mean ± SD)) with no history of diabetes among their parents (Table 1). Additional inclusion criteria of this group were normal levels of plasma lipids, no hypertension, and no obesity, as well as normal hematologic parameters and no alterations in plasma hepatic-related enzymes. These women were submitted to a standard oral glucose tolerance test (OGTT), and blood samples were drawn before (-15 and -5 min) and after (15, 30, 60, and 120 min) the oral glucose (75 g) load. Plasma leptin, adiponectin (total and HMW), and sOb-R levels were measured before (-15 min) OGTT. Plasma concentration of NEFAs, TNF*α*, and MCP1 as well as platelet 5HT levels was measured before (-15 min) and after (120 min) OGTT. Plasma levels of glucose and insulin were measured before and during OGTT. The research protocol of this study was approved by the Institutional Review Board of the School of Medicine, Pontificia Universidad Católica de Chile.

### 2.2. Plasma and Serum Biochemical Determinations

Venous blood samples were obtained at the clinical research facilities of the Red Salud UC (Santiago, Chile) and collected in vacuum tubes using sodium fluoride and EDTA as anticoagulants for glucose measurement and in serum separator gel tubes for insulin measurements. Glucose and insulin levels were measured in the Central Clinical Laboratory at Red Salud UC by the standard colorimetric glucose oxidase method (expressed in mg/dL) and by electrochemiluminescence immunoassay (expressed in *μ*U/mL), respectively. Serum NEFA concentrations were determined by an enzymatic colorimetric method (NEFA-HR, Wako Chemicals, Richmond, VA). Leptin, adiponectin, and c-peptide concentrations were determined by radioimmunoassay (RIA) while sOb-R and high molecular weight (HMW) adiponectin by a commercially available ELISA (R&D Systems, codes: DOBR00 and DHWAD0, respectively). TNF*α* and MCP1 levels were measured by the multiplex technology in a MAGPIX instrument (Merck) using a commercial kit (HMHEMAG-34K). The mean ± SD concentrations of all of these variables are listed in Table 2.

### 2.3. Measurement of Platelet 5HT Levels

Platelet 5HT was measured by means of High-Performance Liquid Chromatography (HPLC) coupled to an electrochemical detector as previously reported [29]. Briefly, platelet-rich plasma (PRP) was obtained from EDTA-blood samples by centrifuging at 200g for 10 min at room temperature. The number of platelets in PRP was counted using a hemocytometer. 5HT was extracted from PRP samples adding perchloric acid (3.4 M), leaving for 30 min in ice for total protein precipitation, and centrifuging at 33,000g for 15 min at 4°C. 5HT mass was estimated by interpolation into a calibration curve and expressed as ng of 5HT by 10^9^ platelets. The HPLC systems consisted of an Ultrasphere 5 *μ*m ODS column (Hichrom, Theale, UK), a Rheodyne manual injector (Sigma-Aldrich, St. Louis, MO), and Waters 515 HPLC pump (Waters, Milford, MA). The HPLC mobile phase was 0.1 M sodium acetate at 1 mL/min. The Waters 464 electrochemical detector was set at 500 mV, a current of 10 nA, and a latency of 5 s using the Empower software (Waters, Milford, MA).

### 2.4. Insulin Secretion/Sensitivity Indexes

Based on plasma glucose and serum insulin levels taken before and during the OGTT, some metabolic indexes were calculated. HOMA-S (the inverse of HOMA-IR (HOMA − S = 1/HOMA − IR)) and the Matsuda ISICOMP were used as surrogates of insulin sensitivity indexes derived from OGTT and calculated as previously reported [30]. By using basal NEFA concentrations, other insulin sensitivity indexes were also calculated to derive Adipose-IR and Revised QUICKI [31]. As surrogate variables of insulin secretion, two indexes were used: the ratio of the total area under the insulin curve relative to the total area under the glucose curve (ratio of AUC insulin/glucose or AUCI/G-R) or relative to the total area under the c-peptide curve (ratio of AUC insulin/c-peptide or AUCR-P-C) [32–35]. Additionally, we calculated the free leptin index (FLI) as the ratio of leptin to the sOb-R concentrations, multiplied by 100 [36].

### 2.5. Statistical Analysis

Associations between fasting platelet 5HT levels and metabolic variables were assessed by Spearman nonparametric correlations and Wilcoxon nonpaired tests. The statistical power estimation for our sample was done considering changes in serum 5HT during OGTT reported by Ho et al. [28]. A sample size of *n* = 59 provides a statistical power of >90% with a confidence of 95% to detect significant changes. Statistical significance was set at *p* < 0.05. STATA 12.0 software (http://www.stata.com) and GraphPad Prism 6 software (https://www.graphpad.com) were used for statistical analysis. The Bonferroni method was applied for multiple comparison correction.

## 3. Results

### 3.1. Platelet 5HT Levels and Adipokine-Related Variables

**Figure 1:**
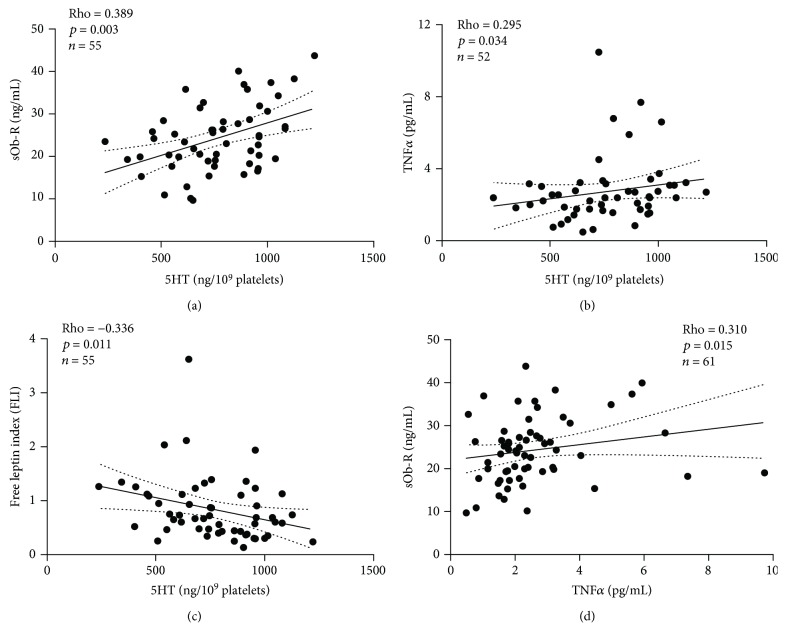
Spearman rank correlation between platelet 5HT and plasma adipokine levels in a cross-sectional sample of Chilean normoglycemic women: (a) soluble leptin receptor (sOb-R); (b) tumor necrosis factor-*α* (TNF*α*); (c) free leptin index (FLI); (d) correlation between platelet 5HT and plasma NEFA concentrations.

In fasted conditions, platelet 5HT levels were directly correlated with plasma sOb-R (rho = 0.39, *p* = 0.003) (Figure 1(a)) and TNF*α* concentrations (rho = 0.30, *p* = 0.03) (Figure 1(b)). Platelet 5HT levels were also negatively correlated with FLI (rho = −0.34, *p* = 0.011) (Figure 1(c)). In turn, we found a positive correlation between TNF*α* and sOb-R (rho = 0.31, *p* = 0.015). Platelet 5HT levels were not associated with other adipokines such as MCP1 and leptin, as well as total or HMW adiponectin (data not shown). Only statistical association between platelet 5HT and plasma sOb-R remained significant after Bonferroni correction of multiple comparisons derived from other adipokines tested in this study (6 tests; corrected *p* = 0.018). Additionally, we found a borderline association between platelet 5HT levels and plasma NEFA concentrations (rho = −0.25, *p* = 0.069) (Figure 1(d)).

### 3.2. Platelet 5HT Levels and Insulin Sensitivity/Secretion Indexes

**Figure 2:**
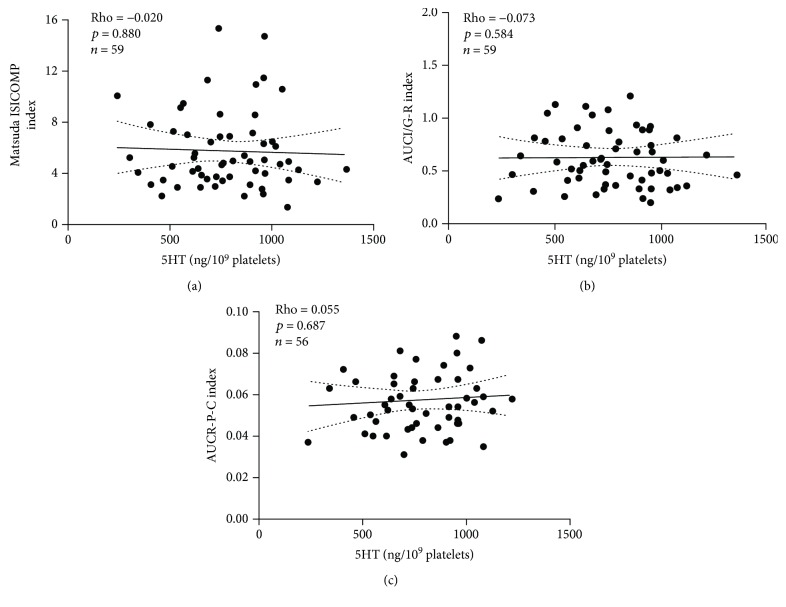
Spearman rank correlation between platelet 5HT levels and insulin sensitivity OGTT-derived indexes in a cross-sectional sample of Chilean normoglycemic women: (a) Matsuda ISICOMP; (b) AUCI/G-R; (c) AUCR-P-C.

We then evaluated whether the fasted platelet 5HT levels were associated with indexes of insulin sensitivity or secretion derived from OGTT in normoglycemic women. We did not find any significant association between the platelet 5HT levels and indexes of insulin sensitivity (Matsuda ISICOMP) (Figure 2(a)). Analysis considering additional insulin sensitivity indexes (HOMA-S, Adipose-IR, and Revised QUICKI) (data not shown) did not show any significant association. We also found no association between platelet 5HT levels and BMI and surrogate indexes of insulin secretion (AUC-IGR and AUCR-P-C) (Figures 2(b) and 2(c)).

### 3.3. Platelet 5HT Levels in response to OGTT

**Figure 3:**
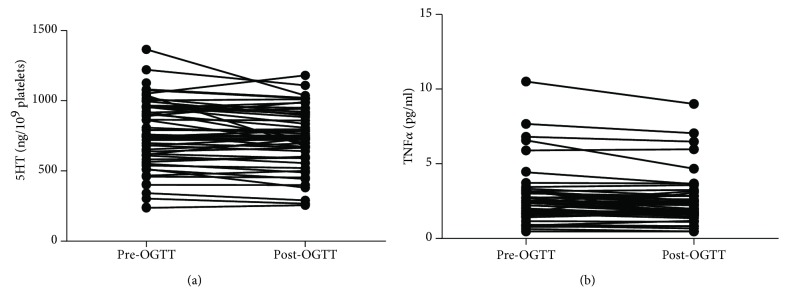
Platelet 5HT and plasma TNF*α* levels before and after 2 h OGTT in a cross-sectional sample of Chilean normoglycemic women.

We evaluated whether platelet 5HT levels changed in response to oral glucose challenge in normoglycemic women. We found a slight but significant decrease in platelet 5HT content adjusted by the platelet number (779 ± 237 vs. 731 ± 217 ng/10^9^ platelets, -15 vs. 120 min, *p* < 0.005) (Figure 3(a)). Additionally, the platelet number was also reduced in response to OGTT (249 ± 59 vs. 238 ± 57 platelets ×1000/mm^3^, *p* < 0.0001). Given the positive correlation between platelet 5HT levels and plasma TNF*α* concentration, we also evaluated levels of this adipokine before and after OGTT. We found a slight but significant reduction of plasma TNF*α* concentration in response to glucose loads in our study group (2.73 ± 1.86 vs. 2.47 ± 1.65 pg/mL, -15 vs. 120 min, *p* = 0.0001) (Figure 3(b)).

## 4. Discussion

There is accumulating evidence indicating that peripheral 5HT plays a physiological role regulating glucose and lipid metabolism and showing the ability 5HT has to modulate hormone secretion on *β*-cells and adipocytes. To gain insight into the physiological role of peripheral 5HT in humans, we have studied the relationship between platelet 5HT levels and metabolic traits.

It is important to take into account that although platelet 5HT represents most of the circulating pool of 5HT (~98%), it is perhaps less representative of the bioactive pool than the free circulating 5HT. Nonetheless, there is no reliable protocol to measure the free circulating 5HT (known as platelet-poor plasma (PPP) 5HT) [37]. Technical artefacts associated with the sample collection and processing, for example, the anticoagulant and antiaggregant agents used as well as the temperature and sheer forces, could dramatically affect the PPP 5HT values (due to platelet activation and releases of high amount of 5HT), and, thus, it is highly not recommended to make conclusions from PPP data [37, 38]. Those technical difficulties have been reflected in a wide range of PPP 5HT reference values reported for humans [37, 39]. Likewise, random changes in PPP 5HT levels, also observed after OGTT in humans, lead to a reasonable doubt in the utility of those measurements [40]. Considering that, we have measured the platelet 5HT instead of PPP 5HT levels in this study.

Leptin and adiponectin are important adipokines regulating energy balance that communicates white adipose tissue with other metabolically relevant tissues such as those of the skeletal muscle, endocrine pancreas, and brain [41, 42]. Imbalances in plasma levels of those hormones are associated with obesity and diabetes [43–45]. In addition, the ratio of leptin/adiponectin (either total or HMW adiponectin) constitutes a useful biomarker of systemic insulin sensitivity [30, 46]. Thus, we first evaluated whether platelet 5HT levels were associated with plasma leptin and adiponectin concentration in normoglycemic women. We did not find significant association between platelet 5HT levels and these adipokines. Our results contrast with a study showing a positive correlation between urine 5HT concentration (as a surrogate of peripheral 5HT) and serum adiponectin concentrations in overweight individuals [47]. The fact that our study was only based on women could not explain the discrepant results, because, in fact, the former study found statistical significance for the mentioned variables in women but not in men [47]. Nonetheless, in contrast to our report, the study population of that investigation was mostly overweight insulin-resistant individuals. Other study has found a reduction in the plasma adiponectin and 5HT levels in dogs subjected to an ad libitum feeding-induced obesity protocol for six months [48]. Thus, it is possible that changes explaining the association between these two hormones are caused by imbalance under overweight/obesity conditions that we were unable to detect in our normoweight study group.

The lack of association between platelet 5HT and plasma adiponectin and leptin levels we found in this study contrasts with a study showing an upregulation of a 5HT receptor signaling cascade in hypertrophic 3T3-L1 adipocytes associated with decreased adiponectin expression [17] as well as with a report showing that 5HT decreases leptin mRNA levels and protein release from rat adipocytes and that subcutaneous injections of 5HT reduced their plasma leptin levels [2]. However, from our study, we cannot exclude a regulatory role of the locally present 5HT in leptin and adiponectin production/release from adipocytes. Although we were unable to determine an association between platelet 5HT levels and plasma leptin and adiponectin concentration, we found a positive correlation between platelet 5HT levels and plasma sOb-R concentrations in normoglycemic women. Our finding may reflect a direct positive regulation of 5HT on sOb-R production or release. In line with our finding, a recent interventional study shows that healthy female volunteers following a protocol to suppress 5HT production (feeding a tryptophan-free diet) have reduced plasma sOb-R concentrations [18]. The sOb-R constitutes the soluble fraction of leptin receptors that regulates the circulating concentration of free leptin and consequently inhibits its actions [49–52]. This is also consistent with the negative correlation we have found between platelet 5HT levels and FLI. Thus, the positive relation between platelet 5HT and plasma sOb-R concentrations found here agrees with previous studies showing that 5HT reduces leptin activity.

The serotonergic and leptin pathways are intrinsically associated within the CNS in the regulation of food intake, energy homeostasis, and glucose and fat metabolism [53, 54]. Due to both leptin and 5HT which can be released by adipocytes [2], it is possible that 5HT interacts not only with leptin pathways in the brain but also with leptin production or release on adipocytes or, even, regulates its bioavailability or bioactivity. In this case, peripheral 5HT may also act as an indirect negative regulator of leptin signaling in the brain. Our findings of the positive correlation between platelet 5HT and plasma sOb-R concentrations and the negative correlation between platelet 5HT levels and FLI seem to agree with this hypothesis, and future studies will need to further investigate this possible interaction between peripheral and central regulation between 5HT and leptin actions.

We then evaluated whether the platelet 5HT levels were associated with plasma concentrations of TNF*α* and MCP1. We found that platelet 5HT levels were positively correlated with plasma TNF*α* concentrations, a proinflammatory adipokine present in higher levels in obese individuals and subjects with insulin resistance [54]. Using *Tph1* (-/-) mice (lacking peripheral 5HT), Duerschmied et al. have shown that 5HT directly stimulates the activity of the TNF*α*-converting enzyme (TACE/ADAM17) [55]. TACE is a sheddase which increases the release of TNF*α* from its inactive cell-bound precursor [54]. This data support the idea that 5HT would be acting as a positive regulator of TNF*α* release.

Similar to TNF*α* levels, the plasma sOb-R levels appear to be increased during inflammatory episodes in humans and animals [56–58]. In addition, treating mice with proinflammatory agents leads to elevated plasma sOb-R concentrations [58]. In line with the reported higher plasma levels of these two proteins in different pathophysiological states, we found a nominal positive correlation between plasma TNF*α* and sOb-R concentrations in normoglycemic women. These positive associations may reflect a common mechanism of regulation of sOb-R and TNF*α* production/release or, alternately, a direct regulation between each other. In support of that hypothetical interaction, *in vitro* analysis has shown that TNF*α* upregulates sOb-R release via ectodomain shedding of long-isoform leptin receptor in several cell types [59]. Thus, considering our data altogether, it is possible to hypothesize a more complex regulatory mechanism in which peripheral 5HT may indirectly enhance sOb-R through the positive regulation of TNF*α*.

In this study, we have also found a reduction of both platelet 5HT content and total platelet count in response to OGTT. In accordance with our result, a study analyzing more than 110 metabolites from 377 nondiabetic individuals in response to OGTT found that plasma 5HT was markedly decreased after glucose load [28]. The magnitude of the change of the circulating 5HT reported in the former study (>2-fold) was much higher than the one reported here. This difference may be due to experimental discrepancies between the former and our study: the characteristic of the study group (insulin resistant vs. healthy women) and the fraction (free vs. platelet 5HT) and method of circulating 5HT quantified (mass spectrometry vs. HPLC) [28]. In contrast with our results, other authors measuring various plasma neurotransmitters in response to OGTT in 100 healthy individuals have found significant increases in platelet 5HT levels [40]. Differences in the group composition (56 vs. 0% males), plasma sample processing, and data calculation may explain our opposed findings; in fact, they have not normalized the 5HT content by the platelet number as it must be. Moreover, these authors have found an increases in platelet 5HT and plasma noradrenalin levels in the individuals that were subjects to a sham-feeding test where they drank a sweetened solution without glucose, meaning the increases of neurotransmitter they observed in response to OGTT may not reflect an effect of the oral glucose load. Other special features of 5HT metabolism could also explain the opposed results, for example, the inter- or intraindividual differences in gut 5HT production or the uptake rate in platelets or in peripheral tissues via SERT (5HT transporter) [60], which could be, in turn, inhibited by the antidepressant drugs (serotonin selective reuptake inhibitor (SSRI)) [61]. The intake of 5HT-rich foods such as walnuts, pineapples, and bananas may also contribute to such interindividual differences in platelet 5HT content [62]. In our study, these interferers were considered exclusion criteria and advertised to participants.

As previously mentioned, there are evidences showing that 5HT acting on *β*-cells inhibits GSIS [3, 7, 21, 22, 24–26, 63–65]. In this study, we have found a reduction of circulating 5HT level in response to glucose load. Thus, a reduction of the inhibitory effect of 5HT signaling in *β*-cells might be necessary for the physiological GSIS, and it would be in line with evidences showing a deregulated 5HT signaling in *β*-cells of subjects with type 2 diabetes (T2D) [3, 66, 67]. However, from our data, we cannot discard the possibility that the observed reduction of platelet 5HT levels in response to OGTT would be a simple consequence of an increased platelet activation in response to the physiological mechanism induced by glucose uptake.

An additional aim of the current study was to assess whether the platelet 5HT levels were associated with OGTT-derivedindexes of insulin sensitivity (Matsuda) or secretion (AUC-IGR and AUCR-P-C). We found no association between platelet 5HT levels and any of these OGTT-derived indexes. These results may reflect that in a physiological range of platelet 5HT levels, such as those found in a cohort of normoglycemic women, the control of peripheral 5HT on insulin sensitivity and secretion may be masked by the multiple metabolic processes potentially regulated by this monoamine. In this context, it has been shown that peripheral 5HT also promotes gluconeogenesis in hepatocytes and negatively regulates energy expenditure by inhibiting the noradrenalin-mediated thermogenesis on brown adipocytes in mice [12, 13]. Therefore, it is probably that imbalance on 5HT local production or free circulating 5HT levels during pathophysiological states, possibly not detected by measuring the platelet 5HT, may contribute to the metabolic disorder progression. In agreement, higher free circulating 5HT but lower platelet 5HT levels has been reported in T2D patients, which has been proposed to be consequences of an increased platelet activation and degranulation under the metabolically adverse low-grade inflammatory states [68–70]. Furthermore, higher plasma and urinary levels of 5-hydroxyindole-3-acetic acid (5-HIAA), a 5HT metabolite, has been associated with metabolic syndrome and diabetes [70, 71].

Thus, the lack of associations between the platelet 5HT and metabolic traits found in this study may be explained by the relatively homogeneous, normoglycemic, mainly normoweight women (with no family history of diabetes) selected for this survey or maybe by the fact that we were measuring the massive amount of platelet 5HT content which is, as previously mentioned, less representative of the bioactive pool than the free 5HT. Additionally, the relatively small sample size (*n* = 59), the lack of information about other risk factors influencing insulin sensitivity such as diet or physical exercise, and the simple observational nature of this study may be considered weaknesses of our research design that may contribute to the decrease in the statistical power of this study.

Therefore, future studies should measure free circulating 5HT (by means of its metabolite 5HIAA) in a large cohort of healthy, glucose-intolerant, and T2D individuals to evaluate whether in a pathophysiological state peripheral 5HT levels explain the differences in insulin sensitivity and/or insulin secretion. Additionally, interventional studies to modify the peripheral 5HT levels, for example, using tryptophan-restricted diet or SSRI drugs, must be performed in humans in order to unveil whether peripheral 5HT is causally associated with alterations of insulin sensitivity or secretion or with appetite regulation.

## 5. Conclusions

We found that platelet 5HT levels are slightly reduced in response to glucose intake and that platelet 5HT levels are positively associated with plasma sOb-R concentrations in normoglycemic women. These findings might reflect a potential role of peripheral 5HT in leptin-mediated appetite regulation.

Our data also indicates that platelet 5HT levels are not associated with indexes of insulin secretion/sensitivity and glycemic-related traits or BMI in this group of Chilean normoglycemic women. Therefore, it is likely that changes in free circulating 5HT or in locally produced key tissues rather than those in platelet 5HT levels explain the reported actions for peripheral 5HT regulating glucose and lipid metabolism.

## Figures and Tables

**Table 1:** Metabolic and anthropometric characteristics of the study population.

Phenotype	Overall (mean ± SD)	*n*	Range
Age (years)	27.2 ± 6.4	59	19-46
Body weight (kg)	60.1 ± 8.4	59	47-91
Height (m)	1.6 ± 0.1	59	1.5-1.7
Body mass index (BMI)	23.6 ± 2.9	59	18.9-33.0
Fasting plasma glucose (mg/dL)	77.6 ± 5.9	58	63-90
Fasting plasma insulin (*μ*U/mL)	8.5 ± 3.6	58	2.3-19.6
2 h glucose (mg/dL)	93.4 ± 20.3	58	50-138
2 h insulin (*μ*U/mL)	65.4 ± 44.2	58	9.3-245.8
HOMA-S index	71.2 ± 28.1	59	25.9-160.5
Matsuda ISICOMP index	5.8 ± 3.0	59	1.38-15.42
Ratio AUC insulin/glucose	0.63 ± 0.3	59	0.2-1.8
Ratio AUC c-peptide/glucose	0.06 ± 0.016	56	0.03-0.10

**Table 2:** Plasma levels of NEFAs, adipokines, and platelet 5HT in the study population.

Phenotype	Overall (mean ± SD)	*n*	Range
NEFAs (-15 min) (*μ*Eq/L)	578.8 ± 189.1	55	116-1034
NEFAs (120 min) (*μ*Eq/L)	104.4 ± 43.5	55	43-230
Leptin (ng/mL)	17.8 ± 8.6	55	4.7-41.3
sOb-R (ng/mL)	24.3 ± 7.7	55	9.7-43.8
Adiponectin (*μ*g/mL)	11.4 ± 4.4	55	2.9-23.3
HMW adiponectin (*μ*g/mL)	3.47 ± 1.47	55	0.1-6.8
TNF*α* (-15 min) (pg/mL)	2.73 ± 1.83	52	0.48-10.5
TNF*α* (120 min) (pg/mL)	2.47 ± 1.65	52	0.47-9.0
MCP1 (-15 min) (pg/mL)	64.1 ± 59.4	54	15.0-432.3
MCP1 (120 min) (pg/mL)	54.1 ± 23.5	54	15.0-149.9
Platelet 5HT (-15 min) (ng/10^9^ platelets)	779 ± 237	59	237-1366
Platelet 5HT (120) (ng/10^9^ platelets)	731 ± 217	52	255-1181

## Data Availability

All the data used in this study are available upon request to the corresponding author.
